# Inducing LTD-Like Effect in the Human Motor Cortex with Low Frequency and Very Short Duration Paired Associative Stimulation: An Exploratory Study

**DOI:** 10.1155/2016/3920298

**Published:** 2016-01-19

**Authors:** Prachaya Srivanitchapoom, Jung E. Park, Nivethida Thirugnanasambandam, Pattamon Panyakaew, Vesper Fe Marie Ramos, Sanjay Pandey, Tianxia Wu, Mark Hallett

**Affiliations:** ^1^Human Motor Control Section, National Institute of Neurological Disorders and Stroke, National Institutes of Health, Bethesda, MD 20892, USA; ^2^Division of Neurology, Department of Medicine, Faculty of Medicine, Siriraj Hospital, Mahidol University, Bangkok 10700, Thailand; ^3^Department of Medicine, Faculty of Medicine, Chulalongkorn Center of Excellence on Parkinson's Disease and Related Disorders, Chulalongkorn University and King Chulalongkorn Memorial Hospital, Thai Red Cross Society, Bangkok 10330, Thailand; ^4^Govind Ballabh Pant Hospital, New Delhi 110002, India; ^5^Clinical Neurosciences Program, National Institute of Neurological Disorders and Stroke, National Institutes of Health, Bethesda, MD 20892, USA

## Abstract

*Introduction.* Paired associative stimulation (PAS) is an established technique to investigate synaptic plasticity in the human motor cortex (M1). Classically, to induce long-term depression- (LTD-) or long-term potentiation-like effects in the human M1, studies have used low frequency and long duration trains of PAS. In the present study, we explored an LTD-like effect using very short duration and low frequency of PAS_10 ms_ protocols in human M1.* Methods.* Six protocols of low frequency PAS_10 ms_ (ranging from 0.2 Hz to 1 Hz) were investigated with very short durations of 1 and 2 minutes stimulation. Six healthy volunteers were included in each protocol. We obtained motor-evoked potentials from right abductor pollicis brevis muscle before and after applying PAS_10 ms_ up to 30 minutes. After we found PAS_10 ms_ protocol which induced an LTD-like effect, we tested that protocol on additional 5 subjects.* Results.* One-way repeated-measures ANOVA showed that only the group of 1-minute stimulation of 0.25 Hz induced an LTD-like effect. When adding the additional subjects, the effect remained and lasted for 30 minutes.* Conclusion.* Low frequency and very short duration of PAS_10 ms_ potentially induced an LTD-like effect in human M1. With further verification, this method might be useful for research relating to synaptic plasticity by reducing the duration of study and minimizing subject discomfort.

## 1. Introduction

Paired associative stimulation (PAS), a method of inducing heterosynaptic plasticity, has been used to investigate bidirectional synaptic plasticity including long-term potentiation- (LTP-) [1–3] and long-term depression- (LTD-) [4–6] like effects in the human motor cortex (M1). The technique of PAS that is frequently used to investigate cortical plasticity is the application of electrical stimulation to the median nerve over the wrist followed by transcranial magnetic stimulation (TMS) over the contralateral M1. Stimulus modalities other than peripheral nerve stimulation have also been used successfully to induce both an LTP- and an LTD-like effect in human M1 [7, 8]. How PAS modulates cortical synaptic plasticity is not exactly known. However, the interstimulus interval (ISI) between median nerve stimulation and motor cortical stimulation appears to be the crucial point to determine the result of the synaptic plasticity, following the concept of spike-time dependent plasticity [4]. Studies showed that the ISI of 10 ms (PAS_10 ms_) could induce an LTD-like effect [4, 5, 9, 10] while the ISI of 25 ms (PAS_25 ms_) could induce an LTP-like effect [1, 2, 4]. To induce an LTD- or an LTP-like effect by using PAS, most studies used low frequency and long duration trains of stimulation [1, 2, 4, 5, 11, 12]. Additionally, Quartarone and colleagues showed that high frequency and very short duration of stimulation (5 Hz of PAS_25 ms_ for 2 minutes) induced an LTP-like effect but failed to induce an LTD-like effect when stimulated with an ISI of 10 ms [13]. To reduce duration of study and the number of the stimuli, we explored whether an LTD-like effect could be induced by using low frequency and very short duration of PAS_10 ms_. Therefore, the objective of our exploratory study was to attempt to induce an LTD-like effect in the human M1 using new PAS_10 ms_ protocols which consisted of low frequency and very short duration of stimulation.

## 2. Materials and Methods

### 2.1. Subjects

Forty healthy volunteers (HVs) (22 females and 18 males, mean age (SD) 36.4 (12.1) years) participated in this study. Eight subjects participated in more than one PAS_10 ms_ protocol experiment. In those 8 subjects, experiments were conducted in the separate sessions with at least 72-hour interval in order to eliminate a possible residual stimulation effect. All subjects were at least 18 years of age, were right-handed by the Edinburgh Handedness Inventory [14], and were certified healthy from a neurological examination. We excluded subjects with history of drugs/alcohol abuse within the past 6 months, taking medications affecting the central nervous system, and with presence of metal or pace-maker implants in the body. Nine subjects were withdrawn because of technical difficulties to obtain the proper motor-evoked potential (MEP) including thick hair, inability to relax their right hand (the investigated hand), continuous moving of their left hand (the contralateral hand), and polyphasic MEP. Those 9 subjects were excluded from the stated 40 subjects and were not included in the statistical analysis. The experiments conformed to the Declaration of Helsinki and were approved by the Institutional Review Board of the National Institute of Neurological Disorders and Stroke, National Institutes of Health. Written informed consent was obtained from all subjects before participation.

### 2.2. Recording

EMG activities were recorded by using disposable surface Ag-AgCl electrodes. Electrodes were placed on the right abductor pollicis brevis (APB) muscle with the active electrode placed over the muscle belly and the inactive electrode placed over the metacarpophalangeal joint of the right thumb. The EMG signal was amplified using a conventional EMG machine (Nihon Kohden Inc., Tokyo, Japan) with bandpass between 10 and 2000 Hz. The signal was digitized at a frequency of 5 kHz and fed into a computer that recorded using Signal software version 5.09 (Cambridge Electronic Design, Cambridge, UK) for offline analysis.

### 2.3. Stimulation

Magnetic stimulation was generated using Magstim super rapid 2 biphasic stimulator (Magstim, Whitland, Dyfed, UK) connected to figure-of-eight coil with an external loop diameter of 90 mm. Stimulation was delivered over the left M1 corresponding to contralateral APB with the coil tangential to the scalp perpendicular to the left M1 and the handle pointed 45-degree posterolaterally for producing the main current in posterior-anterior direction.

Right median nerve stimulation was performed at the wrist through bipolar electrodes with a standard stimulation block (cathode proximal; square-wave with stimulus width 200 *μ*s). The perceptual threshold of median nerve was recorded by application of the minimum intensity of electrical stimuli over the right wrist that were perceived by the subjects.

### 2.4. Experimental Procedures

The experiments were performed during daytime between 10 am to 3 pm in all subjects. Subjects wore a TMS cap and earplugs, sat in a comfortable chair, relaxed their hand, and looked straight ahead and focused on an “X” located 6 feet in front of them. The optimal location of the coil for generating MEP in the right APB was found over the left M1 by using a moderately suprathreshold stimulation intensity, and the location was marked on the cap using a soft-tip pen. At the optimal location, the input-output curve (IOC) parameters were obtained by delivering single TMS pulses at intensities in random order from 5 to 100% (increasing intensity at 5% intervals) of maximal stimulator output with duration of interpulse interval of 5 seconds. Two pulses were delivered at each intensity. Peak-to-peak of MEP amplitudes were recorded and the amplitudes were plotted against the corresponding stimulation intensity. A sigmoid curve was fitted based on the Boltzmann equation. This curve provided the estimated resting motor threshold (RMT) and S50 which is the intensity that can elicit MEP amplitudes equal to 50% of the maximal MEP amplitude. In our study, the S50 intensity generated MEP amplitude of at least 500 *μ*V. Thereafter, the accurate RMT was determined by using the adaptive threshold hunting methodology of Awiszus [15].

In the actual experiment, the intervention consisted of multiple pairs of single electrical stimuli delivered to the right median nerve over the wrist at the intensity of 200% of perceptual threshold followed by the TMS over the left M1 at the intensity of 80% of RMT with the ISI of 10 ms; PAS_10 ms_. We decided to use submotor threshold intensities to avoid sensory reafferent feedback activation caused by muscle twitches. For measuring MEP amplitudes at rest, 20 pulses were delivered before and after intervention using a stimulation intensity of S50 with duration of intertrial interval of 5 seconds. The postintervention MEP amplitudes were assessed at 1 (T1), 5 (T5), 10 (T10), 15 (T15), 20 (T20), 25 (T25), and 30 minutes (T30) after PAS.

**Figure 1:**
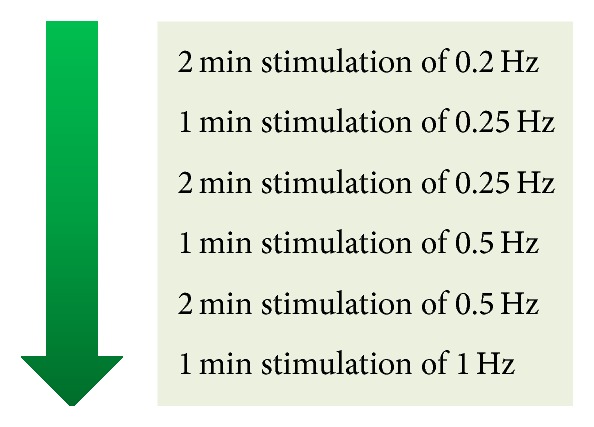
All investigated PAS_10 ms_ protocols. The order of the investigation is following the direction of the arrow which begins with 2-minute stimulation of 0.2 Hz and finishes at 1-minute stimulation of 1 Hz. Six different healthy volunteers were assigned to each protocol by the order of recruitment. We had to complete all 6 subjects in the same protocol before investigating the next protocol. Min = minute and PAS_10 ms_ = paired associative stimulation with interstimulus interval of 10 ms.

According to previous repetitive TMS studies, the low frequency, 0.2 Hz to 1 Hz, could induce inhibitory effect on the human M1 [16]. Therefore, we decided to use low frequency including 0.2, 0.25, 0.5, and 1 Hz for investigating an LTD-like effect. Six groups of PAS_10 ms_ protocol were investigated independently. The order of the investigation is summarized in Figure 1 began with 2-minute stimulation of 0.2 Hz, 1-minute stimulation of 0.25 Hz, 2-minute stimulation of 0.25 Hz, 1-minute stimulation of 0.5 Hz, 2-minute stimulation of 0.5 Hz, and 1-minute stimulation of 1 Hz, respectively. Six different HVs were assigned to each protocol by the order of recruitment. For example, subjects who were recruited as numbers 1 to 6 were assigned to participate in the experiment using 2-minute stimulation of 0.2 Hz, then subjects who were recruited as numbers 7 to 12 were assigned to participate in the next PAS_10 ms_ protocol. We had to complete all 6 subjects in the same protocol before doing an exploratory interim analysis. As a result of the interim analysis, we could add additional subjects and/or move on to investigate the next protocol. After we found a PAS_10 ms_ protocol which significantly induced an LTD-like effect, we tested that protocol on additional 5 subjects and the data were combined for an analysis based on data of 11 subjects.

### 2.5. Statistical Analyses

For each subject, MEP amplitudes from right APB were measured peak-to-peak in each of 20 trials in mV at eight time-points: baseline (preintervention), and T1, T5, T10, T15, T20, T25, and T30 (postintervention). The medians, instead of mean, of 20 trials were calculated for each time-point due to the skewed distributions and were transformed by natural logarithm since the distributions in all six protocols had long right tails.

For each protocol, one-way repeated-measures analysis of variance (RM-ANOVA) was used to evaluate the inhibitory effect, where the factor was the time-point with 7 levels. Each protocol was tested separately to explore the six conditions: 1-minute stimulation of 0.25, 0.5, and 1 Hz and 2-minute stimulation of 0.2, 0.25, and 0.5 Hz. After we identified the PAS_10 ms_ protocol that significantly induced an LTD-like effect, we investigated this particular protocol on additional 5 subjects. The combined data with 11 subjects was also analyzed using one-way RM-ANOVA. Dunnett-Hsu method was applied to* post hoc* analysis with preintervention (baseline) as a control, and Bonferroni correction was used to adjust for multiple testing or protocols. Uncorrected *p* value was multiplied by 6 in the protocols with six subjects and was multiplied by 2 in the combined data (total 11 subjects) considering that only this particular protocol was tested twice. The statistical analyses were performed using SAS version 9.2.

## 3. Results

Baseline characteristics of the subjects in all 6 protocols including the additional 5 subjects in the protocol which induced an LTD-like effect are summarized in Table 1. One-way ANOVA of all baseline characteristics did not show statistical significant difference among 6 protocols. No subjects reported any adverse effects during or after the experiments.

**Figure 2:**
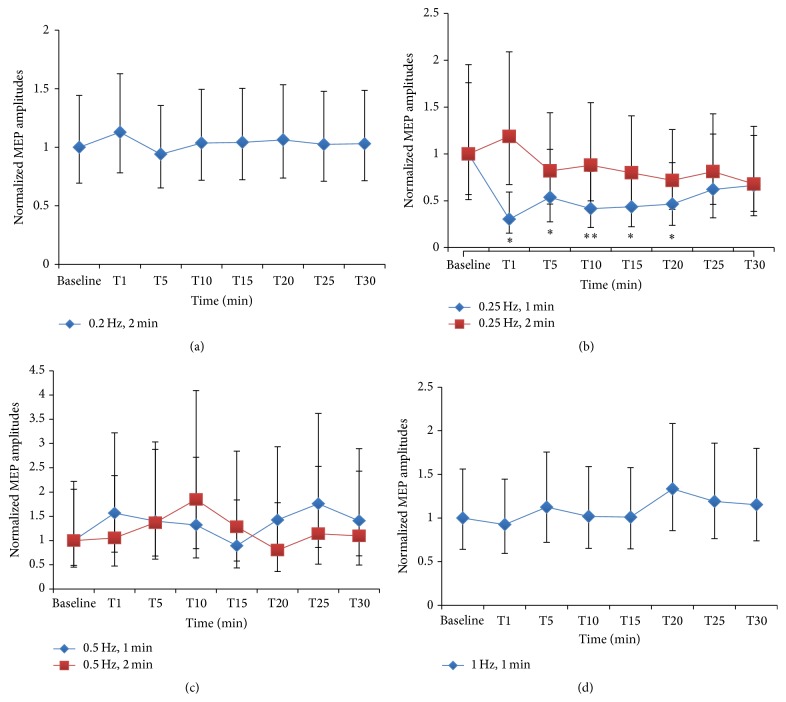
Normalized MEP amplitudes of 6 subjects with 95% confidence interval of each PAS_10 ms_ protocol. One-way repeated-measures ANOVA showed a statistically significant inhibition, an LTD-like effect, of PAS_10 ms_ protocol of 1-minute stimulation of 0.25 Hz (reporting as an uncorrected *p* value) ((b); diamond shape; ^*∗∗*^ *p* value < 0.05).* Post hoc* analysis of this protocol showed that the inhibitory effect (reporting as an uncorrected *p* value) began immediately after applying PAS_10 ms_ (T1), 10 (T10), 15 (T15), and 20 minutes (T20) (^*∗*^ *p* value < 0.05). The maximum inhibition was approximately 70% reduction. The PAS_10 ms_ protocols of 2-minute stimulation of 0.2 Hz (a), 0.25 Hz ((b) square shape), and 0.5 Hz ((c) square shape) and 1-minute stimulation of 0.5 Hz ((c) diamond shape) and 1 Hz (d) did not show either inhibition or facilitation. Min = minute, MEP = motor-evoked potentials, LTD = long-term depression, and PAS_10 ms_ = paired associative stimulation with interstimulus interval of 10 ms.

One-way RM-ANOVA showed a statistically significant inhibitory effect, an LTD-like effect, in the PAS_10 ms_ protocol of 1-minute stimulation of 0.25 Hz (*F* value = 3.42; *p* value = 0.007; Bonferroni adjusted *p* value = 0.04) whereas the other protocols including 2-minute stimulation of 0.2 Hz (*F* value = 0.33; *p* value = 0.94; Bonferroni adjusted *p* value = 5.62), 0.25 Hz (*F* value = 0.95; *p* value = 0.48; Bonferroni adjusted *p* value = 2.90), and 0.5 Hz (*F* value = 1.44; *p* value = 0.22; Bonferroni adjusted *p* value = 1.34) and 1-minute stimulation of 0.5 Hz (*F* value = 1.47; *p* value = 0.21; Bonferroni adjusted *p* value = 1.27) and 1 Hz (*F* value = 0.94; *p* value = 0.49; Bonferroni adjusted *p* value = 2.95) did not show either inhibitory or facilitatory effects.* Post hoc* analysis of 1-minute stimulation of 0.25 Hz of PAS_10 ms_ showed a statistically significant reduction of MEP amplitudes at 1, 10, 15, and 20 minutes after intervention compared with baseline (*p* value = 0.001, 0.02, 0.03, and 0.045, resp.). The maximum inhibitory effect was approximately 70% reduction and the inhibitory effect lasted for 20 minutes. However, after adjusting the *p* value by using Bonferroni correction, statistically significant inhibition was only at 1 minute after intervention (Bonferroni adjusted *p* value = 0.004). Normalized MEP amplitudes of 6 subjects with 95% CI of pre- and postintervention of each PAS_10 ms_ protocol are illustrated in Figure 2.

**Figure 3:**
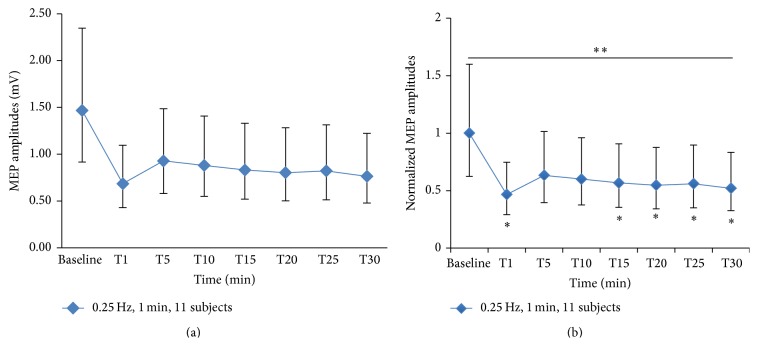
Original MEP amplitude and normalized MEP amplitudes of 11 subjects with 95% confidence interval of 1-minute stimulation of 0.25 Hz PAS_10 ms_. The original MEP amplitudes are reported as a median with 95% confidence interval (a). One-way repeated-measures ANOVA showed a significant LTD-like effect in 11 subjects (reported as an uncorrected *p* value) (^*∗∗*^ *p* value < 0.05).* Post hoc* analysis showed that the inhibitory effect began immediately after applied PAS_10 ms_ (T1), 15 (T15), 20 (T20), 25 (T25), and 30 minutes (T30) (^*∗*^ *p* value < 0.05). The maximum inhibition was approximately 55% reduction (b). Min = minute, MEP = motor-evoked potentials, LTD = long-term depression, and PAS_10 ms_ = paired associative stimulation with interstimulus interval of 10 ms.

**Figure 4:**
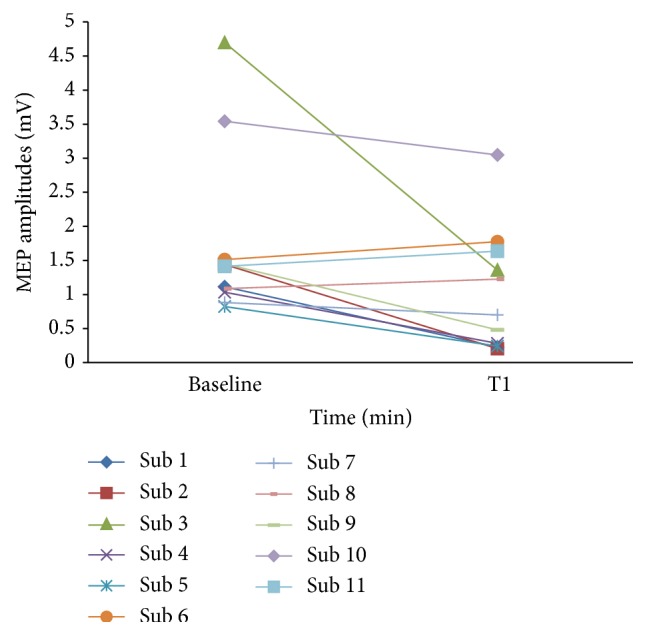
Comparison of the original MEP amplitudes at 1 minute after stimulating with protocol of 1-minute stimulation of 0.25 Hz PAS_10 ms_ with baseline of all 11 subjects. The greatest inhibitory effect was seen at 1 minute after stimulating with the protocol of 1-minute stimulation of 0.25 Hz PAS_10 ms_. Inhibitory effects were found in 8 subjects whereas other 3 subjects showed mild elevation. Sub = subject, MEP = motor-evoked potential, and PAS_10 ms_ = paired associative stimulation with interstimulus interval of 10 ms.

The one-minute stimulation of 0.25 Hz of PAS_10 ms_ protocol was tested on additional 5 subjects and the data were combined with the prior data of 6 subjects then entered into the main analysis again. One-way RM-ANOVA showed statistically significant inhibition (*F* value = 2.47; *p* value = 0.025). However, after adjusting the *p* value by using Bonferroni correction, statistically significant inhibition was marginal (Bonferroni adjusted *p* value = 0.05).* Post hoc* analysis showed a statistically significant reduction of MEP amplitudes at 1, 15, 20, 25, and 30 minutes after intervention compared with baseline (*p* value = 0.003, 0.04, 0.025, 0.03, and 0.01, resp.). The maximum inhibitory effect was approximately 55% reduction and the inhibitory effect lasted for 30 minutes. However, after adjusting the *p* value by using Bonferroni correction, statistically significant inhibition was only at 1 and 30 minutes after intervention (Bonferroni adjusted *p* value = 0.005 and 0.03, resp.). The original MEP amplitudes which are reported as a median and normalized MEP amplitudes of total 11 subjects with 95% CI of pre- and postintervention of 1-minute stimulation of 0.25 Hz of PAS_10 ms_ protocol are illustrated in Figures 2(a) and 2(b), respectively. In addition, the individual data on the MEP amplitudes change at 1 minute after stimulating with 1 minute of 0.25 Hz PAS_10 ms_ protocol which exhibited the greatest inhibitory effect are presented in Figure 3. Comparison of the original MEP amplitudes of all 11 subjects who were investigated with protocol of 1-minute stimulation of 0.25 Hz PAS_10 ms_ between preintervention and immediate postintervention (T1) which revealed the greatest inhibitory effects is showed in Figure 4.

## 4. Discussion

The present exploratory study showed that a new PAS_10 ms_ protocol consisting of low frequency (0.25 Hz) and very short duration (1 minute) of stimulation induced an LTD-like effect in the human M1. To induce an LTD-like effect in the human M1 by using PAS, factors that might be considered include the ISI between median nerve stimulation and M1 stimulation, and the frequency and total duration of paired stimulation. A previous study conducted by Wolters and colleagues showed that an LTD-like effect was induced by stimulating the human M1 with 0.05 Hz of PAS_10 ms_ for 30 minutes whereas an LTP-like effect was produced by using PAS_25 ms_ for 30 minutes [4]. The explanation of these findings is based on the concept of spike-timing dependent plasticity (STDP). Our study also showed that PAS_10 ms_ is able to induce an LTD-like effect in the human M1. Thus, the ISI between median nerve and M1 stimulation seems to be the crucial role to determine the type of motor cortical plasticity. However, the mechanism for modulating synaptic plasticity in human M1 using different protocols of PAS paradigm may not be solely explained by the concept of STDP. For example, recent studies related to PAS inducing an LTP-like effect showed that different timing of afferent input, ISI of 25 ms and 21.5 ms, could induce an LTP-like effect with different network. While PAS_25 ms_ induced an LTP-like effect through the cerebellar network, PAS_21.5 ms_ did not [17, 18]. Therefore, the inhibitory effects resulting from the new paradigm, short duration, low frequency PAS using submotor threshold stimulation, both peripherally and centrally, may be mediated by different mechanisms compared with conventional PAS_10 ms_. The cellular mechanisms underlying the spike-timing dependent depression are less clear. Postsynaptic L-type voltage-gated calcium channels [4], and both ionotropic (N-methyl-d-aspartate) [4] and metabotropic (group 1 mGluRs) glutamatergic receptors [19] might contribute to the mechanism of an LTD-like effect of PAS.

Another factor that may contribute to plastic change is the frequency and total duration of paired stimulation. In conventional PAS, the frequency and total duration of PAS that is used to induce both an LTP- and an LTD-like effect is 0.05 Hz for 30 minutes (total of 90 pairs) [1, 4]. Further studies that induced the LTD-like effect used various frequencies and total duration of PAS_10 ms_. For example, De Beaumont and colleagues conducted a study of bidirectional cortical plasticity in concussed athletes and HVs [11]. In their study, the part that investigated an LTD-like effect, the result showed that significant reduction of the mean of MEP amplitudes of right APB in HVs occurred immediately after 13-minute stimulation of 0.25 Hz PAS_10 ms_ (total of 195 pairs). Another study, conducted by Weise et al., showed that significant reduction of the mean of MEP amplitudes of right APB in HVs appeared around 45–55 minutes after 30-minute stimulation of 0.1 Hz PAS_10 ms_ (total of 180 pairs) [5]. In our study, an LTD-like effect immediately occurred by using stimulation of 0.25 Hz PAS_10 ms_, but for only 1 minute (total of 15 pairs). Indeed, ISI of 10 ms or N20 minus 5 ms [6, 20, 21] between median nerve and M1 stimulation is established to induce an LTD-like effect in the human M1. However, the total number of pairs of PAS that is calculated from the frequency and total duration of stimulation might influence the amount of change of cortical plasticity. Previous studies showed that a greater number of pairs of PAS_25 ms_ could increase the facilitatory effect of an LTP-like effect [3, 22]. Conversely, no study has supported the correlation between the number of pairs of PAS_10 ms_ and the amount of inhibition of an LTD-like effect. Our study showed the new finding that a low number of paired stimulations can also induce an LTD-like effect. The amount of inhibition was approximately 55% and lasted for 30 minutes.

Explaining why our different PAS protocols caused different after-effects is uncertain; however, there are a number of possible hypotheses. First, the configuration of TMS waveform used in this study was biphasic. A monophasic waveform stimulates postsynaptic neurons in a single direction while a biphasic waveform stimulates postsynaptic neurons in both directions. A previous study showed that stimulation of human M1 with a biphasic waveform of repetitive TMS at 5 Hz induced marked facilitatory effects and induced less facilitation when stimulated at relatively slow frequency such as 1 Hz [23]. Conversely, stimulation with monophasic waveform at a similar frequency did not exhibit a facilitatory effect [23]. According to this result, we postulate that the biphasic waveform may predominantly stimulate excitatory neurons compared with inhibitory neurons. Therefore, if we stimulate M1 with a frequency close to 1 Hz, there may be only little net effect of the stimulation due to a close balance between facilitation and inhibition compared with lower frequencies such as 0.25 Hz which may yield inhibition. The results in this present study are compatible with this proposal since stimulation at frequencies of 0.5 Hz and 1 Hz did not show either facilitation or inhibition while stimulation with 0.25 Hz showed inhibition. Our results also showed that stimulation with 0.2 Hz PAS_10 ms_ did not induce either an LTP- or an LTD-like effect. In this regard, we argue that the stimulation protocol itself was not sufficient to alter synaptic plasticity.

A second possibility is that some subjects who participated in the protocols who did not have either facilitation or inhibition might be classified as “nonresponders” to PAS. Indeed, 61% of healthy subjects can be considered as nonresponders after stimulation with 0.25 Hz PAS_25 ms_, since they did not exhibit an LTP-like effect [24]. In addition, nonresponders to PAS_25 ms_ showed a higher amount of short intracortical inhibition (SICI) before PAS protocol compared with responders group. There have been no reports related to responders and nonresponders in PAS-LTD protocols.

A third factor that might influence cortical plasticity is the brain-derived neurotrophic factor (BDNF) gene. Cheeran and colleagues conducted a study on the effects of various types of noninvasive brain stimulation on HVs who had the Val66Met polymorphism of the BDNF gene. The stimulation included homosynaptic stimulation, for example, continuous theta-burst stimulation (cTBS), which can induce an LTD-like effect, and intermittent TBS (iTBS), which can induce an LTP-like effect, and heterosynaptic stimulation with 0.25 Hz PAS_25 ms_ which also can induce an LTP-like effect [25]. The results showed that the BDNF Val66Met allele was associated with smaller amount of cortical plasticity changes in both an LTD- and an LTP-like effect protocols after applied cTBS and iTBS, respectively, but it did not show any modulation of cortical plasticity when PAS_25 ms_ protocol was applied to subjects. The study concluded that BDNF Val66Met might be a factor influencing the capability of cortical plasticity. However, the study did not include a PAS-LTD protocol. Therefore, at this point, we cannot draw the conclusion that all the subjects who did not show either inhibitory or facilitatory effects in our study should be considered as a nonresponder to PAS.

Fourth, using low-intensity, submotor threshold stimulation, both peripherally and centrally, may stimulate different sets of cortical neurons compared with intensity at supramotor threshold. While intensity at supramotor threshold may stimulate fast-conducting corticospinal output cells, intensity at submotor threshold may stimulate cortical interneurons which subsequently activate the corticospinal output neurons [13]. We postulate that, in our study, the TMS induced postsynaptic activity in the cortical interneurons and the median nerve stimulation generated presynaptic activity by activation of sensorimotor inputs onto these interneurons.

Moreover, to explain why an LTD-like effect could be induced with 1 but not 2 minutes of stimulation of 0.25 Hz protocol is challenging, and we cannot be certain. However, considering the molecular basis of Ca^2+^ influx at postsynaptic neurons, there may be a limit of the amount of Ca^2+^ flowing to postsynaptic neurons that is able to facilitate synaptic plasticity. If the amount of Ca^2+^ flowing into the neurons is greater than this limit, the alteration of synaptic plasticity may not occur. Therefore, we postulate that the 2-minute stimulation may provide a damaging excess of Ca^2+^ preventing the development of an LTD-like effect.

Further, we acknowledge our study's limitations, being an exploratory study that involved small numbers of subjects in each protocol. It would be valuable in the future to investigate this particular PAS_10 ms_ protocol in a larger population along with preintervention SICI and blood testing for* BDNF* Val66Met to identify the possibility of nonresponders to PAS. In conclusion, our finding is promising and might be useful for future research related to investigation of synaptic plasticity by reducing the duration of experiments and minimizing subject discomfort and fatigue with fewer stimulations.

## Figures and Tables

**Table 1:** Baseline characteristics of all PAS_10 ms_ protocols^*∗*^.

	0.2 Hz 2 min	0.25 Hz 1 min		0.25 Hz 2 min	0.5 Hz 1 min	0.5 Hz 2 min	1 Hz 1 min
		Prior 6 subjects	Total of 11 subjects^+^				
Number of pairs	24	15	15	30	30	60	60
Age (years; mean ± SD)	45.50 ± 14.14	34.33 ± 8.87	35.82 ± 10.90	36.67 ± 11.79	28.67 ± 3.98	35.67 ± 14.72	34 ± 7.69
RMT (%; mean ± SD)	59.67 ± 7.71	54.17 ± 8.38	57.73 ± 7.73	56.17 ± 8.47	63.50 ± 16.02	56.83 ± 9.93	57.00 ± 10.56
S50 (%; mean ± SD)	76.67 ± 9.27	70.00 ± 12.68	72.18 ± 10.01	66.83 ± 8.30	78.50 ± 11.33	67.50 ± 12.69	80.17 ± 12.17
200% perceptual threshold (mA; mean ± SD)	7.60 ± 4.09	8.80 ± 2.49	7.78 ± 2.51	9.40 ± 0.83	7.20 ± 2.01	7.53 ± 4.27	6.67 ± 1.95

^*∗*^One-way ANOVA did not show statistically significant difference compared to each baseline characteristics among 6 protocols (*p*-value < 0.05) and ^+^combined additional 5 subjects to prior 6 subjects. PAS_10 ms_ = paired associative stimulation at interstimulus interval of 10 ms, min = minute(s), SD = standard deviation, RMT = resting motor threshold, S50 = the intensity that can elicit MEP amplitudes equal to 50% of the maximal MEP, perceptual threshold = intensity of electrical stimuli at the median nerve over right wrist area that triggered subjects to start feeling the stimulation, and mA = milliamp.
